# Enhancements on Flame Resistance by Inorganic Silicate-Based Intumescent Coating Materials

**DOI:** 10.3390/ma14216628

**Published:** 2021-11-03

**Authors:** Sin-Nan Chen, Pei-Kai Li, Tar-Hwa Hsieh, Ko-Shan Ho, Yu-Meng Hong

**Affiliations:** 1 Department of Chemical and Materials Engineering, National Kaohsiung University of Science and Technology, Kaohsiung City 807618, Taiwan; n58001222@gmail.com (S.-N.C.); oiu16200@gmail.com (P.-K.L.); 2 Department of Chemistry, National Cheng Kung University, No. 1, University Road, Tainan 70101, Taiwan; uytiuyti2001@yahoo.com.tw

**Keywords:** inorganic silicate-based intumescent flame-resistance coating, sodium silicate, metakaoline, expandable graphite, geopolymer, complex flame-resistance layer, carbon-char layer, silicon dioxide networks, fire rating

## Abstract

Flame-retardant coatings have drawn much attention in recent years. In this study, an inorganic sodium silicate-based intumescent flame-resistance coating with an excellent flameproof properties is developed by mainly utilizing sodium silicate as the ceramizable binder, via hydrolysis and self-condensation reaction. Fly ash, metakaoline, and wollastonite behave as supplement cementing materials. Major formulation encompasses the combination of the ammonium polyphosphate and pentaerythritol as the flame-retardant additives, and aluminum hydroxide or expandable graphite as the intumescence-improving filler agents. Expandable graphite was found to play an important role in the eventual performance of flame-resistance testing. The results showed that solid interaction forces can be formed between metakaoline and sodium silicate, resulting in a similar material to geopolymer with excellent physical properties. After high-temperature flame testing, a densely complex protective layer of carbon-char created on top of the robust silicon dioxide networks offers notable flame resistance. An optimal ratio in this inorganic intumescent coating contains sodium silicate—metakaoline (weight ratio = 9:1)—ammonium polyphosphate and pentaerythritol, aluminum hydroxide (3, 3, 10 wt.%)—expandable graphite (1 wt.%), which can create 4.7 times higher expansion ratio compared with neat sodium silicate matrix. The results of flame testing demonstrate only 387.1 °C and 506.3 °C on the back surface of steel substrate after one and three hours flaming (>1000 °C) on the other surface, respectively, which could meet the requirements according to the level of fire rating.

## 1. Introduction

As notable progress has been made in flame-retarding abilities in recent years, four types of flame-retarding materials or approaches have been developed, including flame quencher, heat absorber [1], intumescent flame retardance [2] and synergist [3,4]. Among these, intumescent flame retardance is one of the promising ways and effective methods of protecting substrate from fire damage. A number of organic intumescent materials related to flame retardancies [5,6,7,8,9,10,11] have been published. However, inorganic counterparts are still rarely adopted as the flame-retarding materials, owing to the lack of understanding of the expansion properties of inorganic binder. Usually, organic resins are vulnerable to high-temperature and thermal attacks or even serious drawbacks such as the emission of volatile organic gases, toxic and corrosive fumes [12]. Therefore, inorganic intumescent coatings [13], especially for silicate-based, have gradually emerged as a potential alternative discipline. Sodium silicate-based composite is broadly used in building construction, and can be synthesized through hydrolysis and condensation reactions to build ceramic -Si-O-Si- frameworks at high temperatures. Their physical properties are heavily dependent on the pH variations [14,15] and the presence of curing agents [16], especially when considering workability, mechanical strength and durability improvements.

The inorganic sodium silicate-based flame-retardancy coating has many merits, for example, its moderate harness, low thermal conductance, high anticorrosive resistance, better aging-resistant abilities and increased durability [17]. However, the inability to perform as a flame-resistant material at low temperatures, inferior mechanical strength, and poor adhesion with protected matters are major drawbacks, and largely restrict its application. The introduction of appropriate amounts of supplementary cementing materials, flame retardants and expandable fillers into this system can improve the physical properties or characteristics of inorganic sodium silicate-based flame-retardancy coating materials, for example, by increasing the pull-off strength [18,19], temperature resistance [20,21,22], flame resistance [23,24], and melting temperature [25,26].

As many supplementary cementing materials are available on the earth, we are able to use these minerals as additives in sodium silicate systems to form materials such as geopolymers, with excellent properties, e.g., a high compressive strength, low shrinkage, high-temperature resistance [27], acidic resistance, and flame resistance [28]. The commonly studied microstructure formation, evolved from pore microstructure, is to enhance the compressive strength and thermal stability between geopolymer and Portland cement. However, geopolymer can possess superior properties if the voids are filled by hydration of the input silicates [29]. Zhang et al. [30] demonstrates that the addition of some fly ash can gradually enhance the mechanical and thermal properties of geopolymer until 10 wt.% loading, and significant improvements were found when wt.% was over 10%. The research showed the ratio of sodium silicate to NaOH and is a crucial parameter in controlling the mechanical properties of geopolymer where metakaolin is included. The interpretation is that, when more bonding is created through Si-OH hydrolysis and condensation, its mechanical strength is correspondingly increased [31]. Research indicates that the significant improvements in the ductility of geopolymer are related to the addition of wollastonite, due to the shape effect [32]. Alternatively, traditional flame-retarding materials that evolved into intumescent coating materials have become the mainstream approach, where three basic ingredients, including a flow agent, acid-catalyzed source and charring agent, are incorporated [8]. During flame exposure, a protective carbon-char layer can be created via complex reactions, which prevents inner materials from direct contact with flame. To maintain the steady supply of gases necessary to expand the materials, some metal oxide, such as Al(OH)_3_ or Mg(OH)_2_, are required [1]. These metal oxides are not only able to absorb heat but also release gas or water stream, which can reinforce the flame resistance. With the aforementioned components, only flame-retarding is still insufficient to induce the large area-temperature drop during fire. This is due to the need for more gas expansion as a heat-insulating space to absorb heat. Therefore, expandable graphite with a layer-by-layer structure in whichacidic gas is embedded can effectively solve the expansion problem [33,34].

Since few studies have concentrated on inorganic flame-retardant coating, we aim to provide a new kind of inorganic material to replace the organic-based one. Ceramic sodium silicate is a major matter with high tolerance of flame invasion. Others, such as Metakaolin, fly ash and wollastonite, are supplementary cementing materials with the purpose of forming solidified geopolymer-like materials. Ammonium polyphosphate and pentaerythritol can be responsible for carbon-char formation and protecting the top coating. Aluminum hydroxide and expandable graphite are propellers that can improve the flame-retardancy performance. Eventually, this expandable ceramic-based fire-resistance coating material can pave the way towards many applications in the fields of aerospace, building construction and infrastructure.

## 2. Materials and Methods

### 2.1. Preparations of Sodium Silicate Intumescent Flame-Resistance Coating

Firstly, phenyltrimethylsilane (purity = 94%) was dispersed into water at 50 °C with fixed ratio, stirred for an hour until the mixture became uniform, and then cooled to room temperature. Secondly, sodium silicate (modulus: 32, extra pure reagent) powders with different weight ratios to supplementary cementing materials, such as metakaolin (SiO_2_ 54 ± 2%, Al_2_O_3_ 43 ± 2%), fly ash (SiO_2_ 52.23%, Al_2_O_3_ 27.55%, Fe_2_O_3_ 9.65%, CaO 6.23%, TiO_2_ 1.55%, MgO 1.27%), and wollastonite (SiO_2_ 50.77%, CaO 45.26%, MgO 1.29%), were mixed together. The mixture was stirred until homogeneous by a mechanical stirrer at 250 rpm to form a uniform powder mixture. In a separate reactor, the prepared phenyltrimethylsilane solution was then mixed with the prepared powder mixture and stirred to form a homogeneous sodium silicate-based geopolymer paste. Eventually, the prepared geopolymer paste was cured in the mold in the ambient for a week, forming solidified sodium silicate-based geopolymer material.

To prepare appropriate intumescent flame-resistance coatings, flame-retardant fillers (ammonium polyphosphate (purity = 98%)), pentaerythritol (purity = 99%), aluminum hydroxide (purity > 99%, particle size = 52 μm (behaving as heat absorber and water steam supplier), and expandable graphite (carbon content > 99%, particle size = 270 μm, intumescent improver) were mixed with sodium silicate-based geopolymer paste at various weight ratios and stirred at room temperature, followed by drying for a week in ambient atmosphere. Finally, a formulated intumescent coating was brushed on a steel panel to create a layer with thickness of 5 mm, and underwent further characterization and flame exposure testing.

### 2.2. Characterization and Measurements

Surface morphologies of intumescent flame-resistance coatings were observed by scanning electron microscopy. X-ray diffractometer Bruker, D8Advance was used to analyze the crystalline structure of intumescent flame-resistance coatings. Thermogravimetric analyzer TA Instrument, SDT 2960 Simultaneous DTA-TGA performed thermal stability analysis. The expansion ratios of intumescent flame-resistance coatings were calculated by Archimedes method along with the variation in volume. The corresponding mechanical properties, such as hardness and pull-off strength onto steel substrate, were measured with Shore D hardness tester and adhesion meters (based on the standard of EN 1542), respectively. To testify flame resistance, intumescent flame-resistance coatings adhered onto steel substrate were combusted by pilot flame (>1000 °C) at a distance of ~10 cm.

## 3. Results and Discussion

### 3.1. Properties of Sodium Silicate-Based Intumescent Geopolymer Materials

Intumescent sodium silicate-based materials play a major role by improving the expansion ratio to inhibit thermal conduction and follow flaming, which is one of the key mechanisms for flame resistance. The -Si-O-Si- networks are formed through hydrolysis and condensation reactions in the sodium silicate matrix. The reactions are as follows:

(i) Hydrolysis reaction

Na_2_SiO_3_ → 2Na^+^ + SiO_3_^2−^

SiO_3_^2−^ + 2H^+^ → H_2_SiO_3_

H_2_SiO_3_ + H_2_O → Si(OH)_4_

(ii) Self-condensation reaction

(OH)_4_Si + Si(OH)_4_ → (HO)_3_-Si-O-Si(OH)_3_ + H_2_O

**Figure 1:**
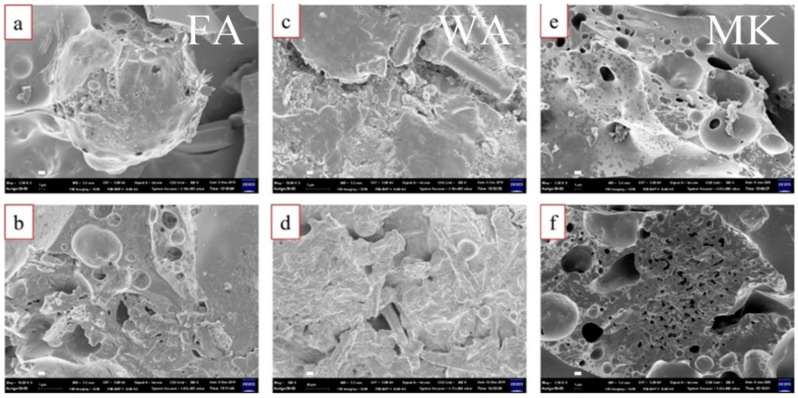
SEM images of sodium silicate-based intumescent materials with 9:1 of sodium silicate matrix to (**a**) fly ash, (**c**) wollastonite and (**e**) metakaolin after flame testing, respectively. The corresponding magnified morphologies (**b**), (**d**) and (**f**), respectively. (scale bar = 1 μm).

**Figure 2:**
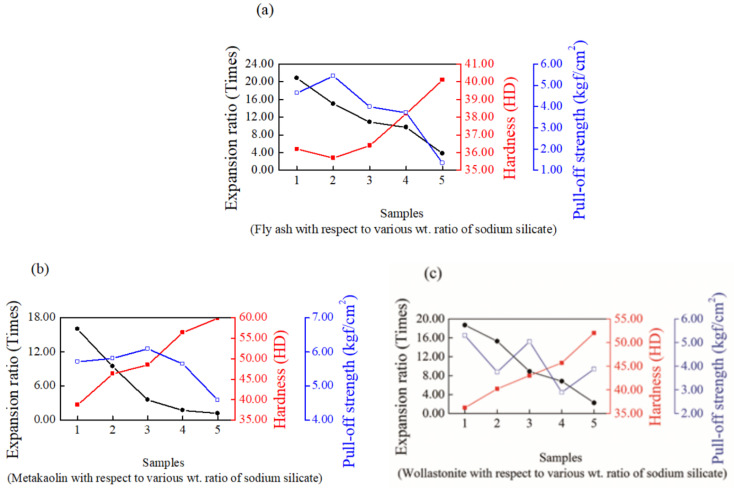
Physical properties of expansion ratio, hardness and pull-off strength of geopolymers containing (**a**) fly ash, (**b**) metakaolin and (**c**) wollastonite at various weight ratios to sodium silicate matrix.

Figure 1 shows the SEM images of intumescent flame-resistance coating consisting of three kinds of supplementary cementing additives at a ratio of 9:1 (the optimal ratio) by weight after flame testing, respectively. As we can see, fly ash (Figure 1a, large sphere-shaped) and wollastonite (Figure 1c, long, fiber-shaped) demonstrate significant unfavorable dispersion morphologies in the intumescent sodium silicate matrix compared to of metakaolin (Figure 1e, small, sphere-shaped). This suggests that metakaolin can be more uniformly dispersed to further build up the robust network of an intumescent sodium silicate-based geopolymer. Hence, the shape and size of the supplementary cementing materials can influence their bonding conditions with sodium silicate matrix during the construction of the network matrix. Then, we can also change the expansion ratio, hardness, and pull-off strength of physical properties of sodium silicate matrix with the composition of fly ash, wollastonite and metakaolin to obtain the optimal intumescent sodium silicate materials. The addition of supplementary cementing materials to sodium silicate can alter the physical properties of intumescent sodium silicate matrix. Figure 2 shows that the expansion ratio of the intumescent sodium silicate matrix decreases with increasing contents of supplementary cementing materials. The highest and lowest expansion ratios for the fly ash and metakaolin were 20.8 and 16.0 times, respectively. The highest expansion ratio of fly ash mainly came from its larger sphere-like shape, resulting in weaker bonding with the intumescent sodium silicate and a greater increase in expansion ratio. Wollastonite primarily containing CaSiO_3_ gave rise to an intermediate expansion ratio staying between fly ash and metakaolin. Similarly, metakaolin appeared to be the hardest material among the supplementary cementing materials due to its having strongest bonding with sodium silicate (degree of dispersion in the order: metakaolin > wollastonite > fly ash). It also showed a fine spherical shape with a higher packing density. Alternatively, the pull-off strength for the different additives in the intumescent sodium silicate materials was in the order of metakaolin > wollastonite > fly ash. The bonding between sodium silicate materials and steel substrate basically resulted from the reaction of -Si(OH) with the surface iron of steel surface; hence, a -Si-O-Fe bond [35] could be created to govern the adhesion ability. Eventually, the resulting optimal ratio for sodium silicate-based intumescent materials was 9:1 by weight.

### 3.2. Effects of Ammonium Polyphosphate and Pentaerythritol on the Physical and Thermal Properties of Intumescent Flame-Resistance Coating Materials

**Figure 3:**
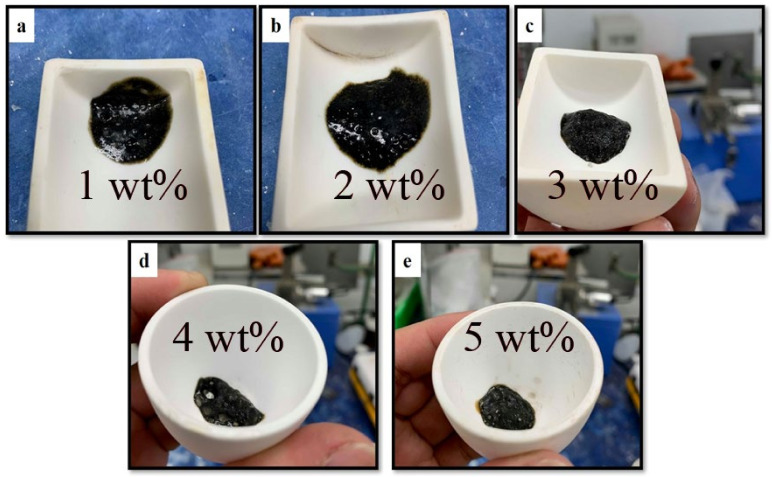
Photographs of sodium silicate-based intumescent flame-resistance coatings with different contents of (**a**) 1, (**b**) 2, (**c**) 3, (**d**) 4 and (**e**) 5 wt.% of ammonium polyphosphate and pentaerythritol after exposure to 1000 °C heating for an hour, respectively.

Due to the relatively low-melting point of foamed sodium silicate matrix (<1000 °C), it cannot tolerate common flame testing which is usually carried out above 1200 °C. Consequently, carbon-based flame-retardant fillers containing ammonium polyphosphate and pentaerythritol are applied to reinforce the high-temperature resistance. Figure 3 exhibits visual images of several composites containing sodium silicate matrix and metakaolin at a weight ratio of 9:1 plus 1~5 wt.% of ammonium polyphosphate and pentaerythritol additives subject to 1000 °C heating for an hour. At a ratio of 3 wt.% (in Figure 3c), intumescent flame-resistance coating maintains structural stability without causing any significant damage to the matrix, suggesting that acid-catalyzed carbon sources can produce a protective flame-resistance layer once exposed to high-temperature flaming, and prevent the foamed sodium silicate binder from further collapse. Intumescent materials carry out some traditional chemical reactions, illustrated in the following:

(i) The pyrolysis of ammonium polyphosphate,

>250 °C

(NH_4_PO_3_)_n_ ------------ > (HPO_3_)_n_ (i)

-nNH_3_

(ii) Carbonized char ([-C-]_x_) from pentaerythritol (Cx(H_2_O)_m_),

(HPO_3_)_n_ + C_x_(H_2_O)_m_ → [-C-]_x_ + (HPO_3_)_n_ x m(H_2_O) (ii),

and (iii) release of water stream for thermal duration acts as part of the blowing source in the sodium silicate matrix due to its self-curing progress without melamine.

∆

H_2_O_(l)_ –––→ H_2_O_(g)_

**Figure 4:**
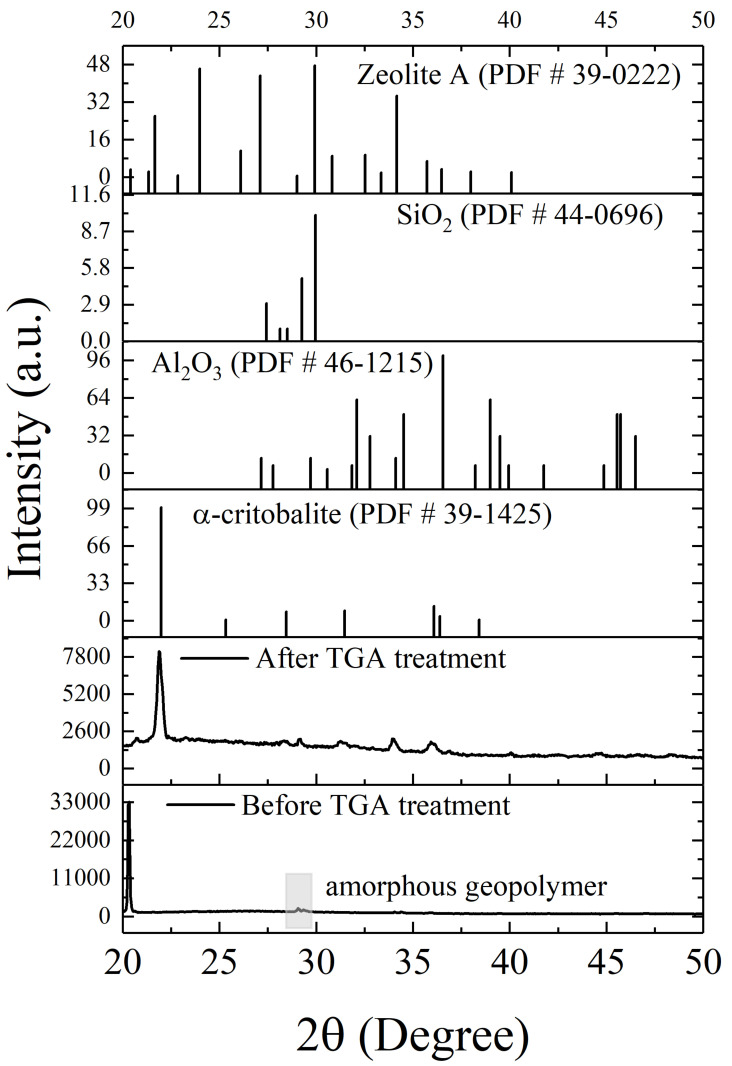
XRD patterns of intumescent flame-resistance coatings with 3 wt.% of ammonium polyphosphate and pentaerythritol before and after thermal treatment in the TGA process.

To verify whether phase change occurs at an elevated temperature, samples experiencing the thermal heating in the TGA process were taken for XRD diffraction, as shown in Figure 4. The major diffraction peak for Na_3_PO_4_·H_2_O at 2θ = 20.3° can be found. This is the product of the reaction between ammonium polyphosphate and the neat (unreacted) sodium silicate matrix during the initial stage of high-temperature heating. Intriguingly, after the thermal treatment in the TGA process, this diffraction peak disappears and a new crystalline phase, named α-cristobalite (JCPDS-PDF #39-1425), was created, indexed at 2θ = 21.98°, 28.43° and 36.07°, respectively. This crystalline inorganic substance and silicon and aluminum oxides and Zeolites A (JCPDS-PDF #44-0696, JCPDS-PDF #46-1215 and JCPDS-PDF #39-0222) obtained from high-temperature calcination might result from the bonding between metakaolin and sodium silicate during the high-temperature ceramic-forming process. This is indirectly in agreement with the results in Figure 3c, which indicates that high-temperature thermal heating not only increases the tendency towards the formation of -Si-O-Al bonding for aluminosilicate geopolymer, but can also improve the related mechanical/physical properties of intumescent flame-resistance coatings.

**Figure 5:**
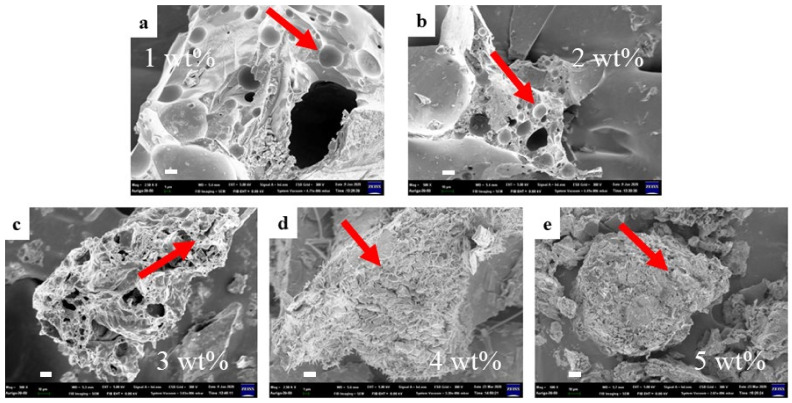
SEM images of intumescent flame-resistance coatings containing sodium silicate with (**a**) 1, (**b**) 2, (**c**) 3, (**d**) 4 to (**e**) 5 wt.% of ammonium polyphosphate and pentaerythritol after TGA’s heating, respectively. (scale bar = 10 μm).

**Figure 6:**
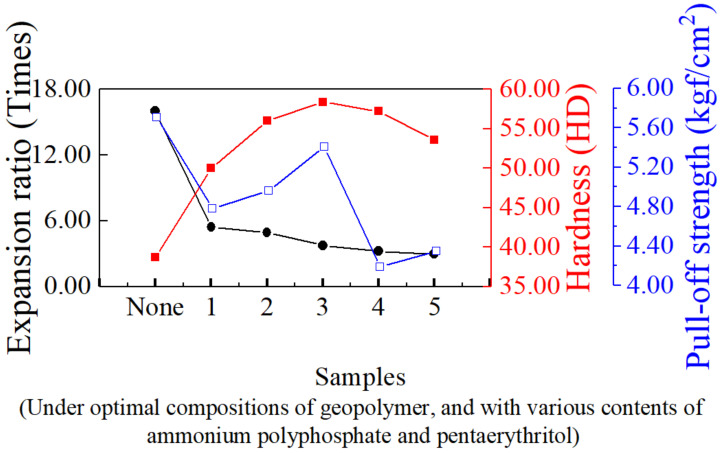
Physical properties of intumescent flame-resistance coatings containing optimal ratio of sodium silicate, ammonium polyphosphate and pentaerythritol after flame testing.

**Figure 7:**
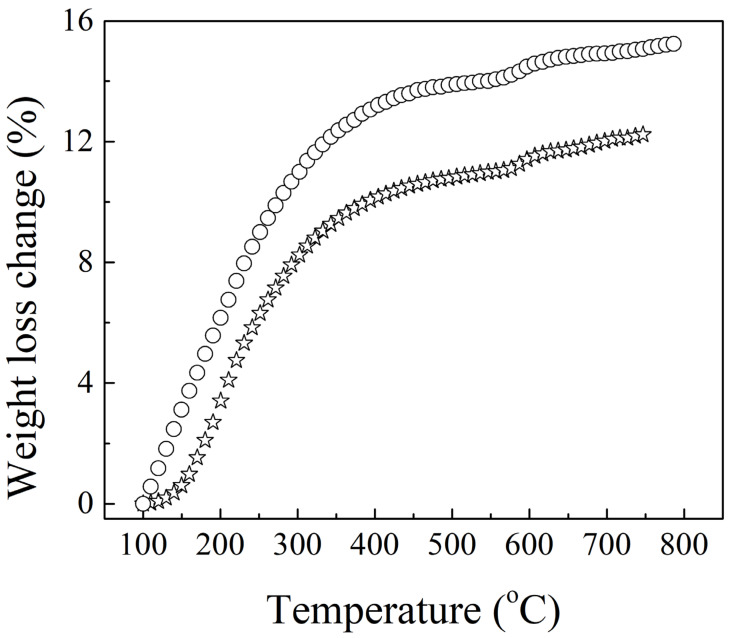
Thermal analysis of intumescent flame-resistance coatings containing neat sodium silicates-based geopolymer (empty circle) and geopolymer with 3 wt.% of ammonium polyphosphate and pentaerythritol (empty star).

To investigate the influence of the presence of both ammonium polyphosphate and pentaerythritol on the properties of intumescent flame-resistance coating, SEM micrographs were taken and shown in Figure 5, which illustrates the pore formation in the passive protective carbon-char layer with various ammonium polyphosphate and pentaerythritol contents. It can be seen that increases in ammonium polyphosphate and pentaerythritol result in a smaller pore size. This implies that many -Si-O-Si- bonds are created to build more robust frameworks. Therefore, increased ammonium polyphosphate could likely trigger more complete solidification reaction with decreasing pH value due, to more -OH being formed along the -O-Si-O- main chain. The phenomenon of expansion ratio decreases with increasing amounts of ammonium polyphosphate due to the enhanced structural stability, as shown in Figure 6. Additionally, the hardness of intumescent flame-resistance coating materials after flame testing was found to achieve the highest value of 58.4 HD with only 3 wt.% of ammonium polyphosphate and pentaerythritol, whereas it gradually decays. When the compositions are over 3 wt.%, the produced Na_3_PO_4_ hydrates are detrimental to the development of three-dimensional network, resulting in a decline in hardness. Regarding pull-off strength, the addition of a small amount of ammonium polyphosphate causes it to drop abruptly, followed by an increase. This likely correlates with the formation of interactions among ammonium polyphosphate and the components inside the coating materials. To understanding the weight lost with temperature, Figure 7 exhibits the thermograms of the geopolymer with and without the addition of 3 wt.% of ammonium polyphosphate and pentaerythritol. Evidently, with the incorporation of ammonium polyphosphate and pentaerythritol, less weight was found than in the absence of ammonium polyphosphate and pentaerythritol. The main thermal degradations occurred at 100~400 °C and 400~600 °C, respectively, in two stages. The first stage is due to the thermal solidification within the sodium silicate matrix, leaving water steam to evaporate during the dehydration–condensation reaction. The reaction of Na_3_PO_4_ with ammonium polyphosphate probably led to the evolution of water vapor and CO_2_ gas, which escaped more significantly at further high-temperature calcination into the next stage, accompanying the pyrolysis of phenyltrimethylsilane. These results indicate that the formation of carbon char can significantly boost flame-resistance.

### 3.3. Effects of Al(OH)_3_ on the Physical and Thermal Properties of Intumescent Flame-Resistance Coating Materials

**Figure 8:**
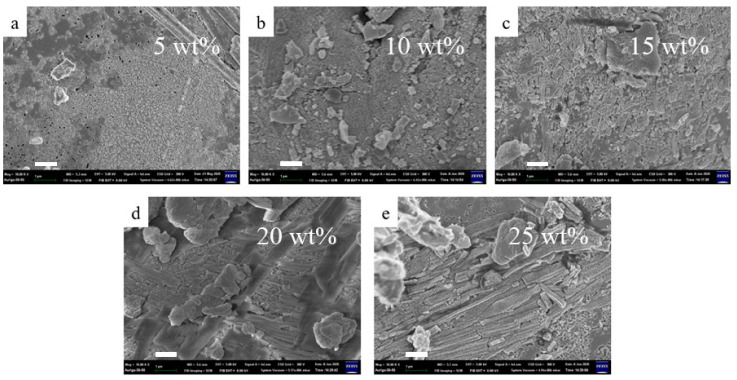
SEM images of intumescent flame-resistant coatings after thermal heating with the optimum ratio of sodium silicate, ammonium polyphosphate, pentaerythritol, plus (**a**) 5, (**b**) 10, (**c**) 15, (**d**) 20 and (**e**) 25 wt.% of Al(OH)_3_, respectively. (scale bar = 1 μm).

**Figure 9:**
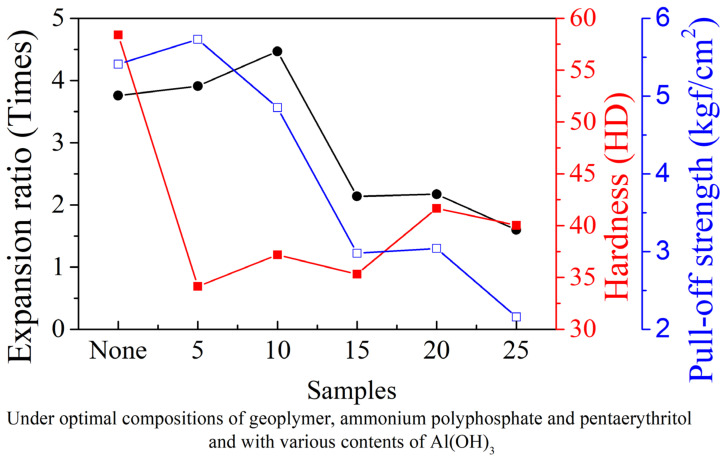
Physical properties of intumescent flame-resistant coatings after flame testing with optimal ratio of sodium silicate, ammonium polyphosphate, pentaerythritol and various contents of Al(OH)_3_.

**Figure 10:**
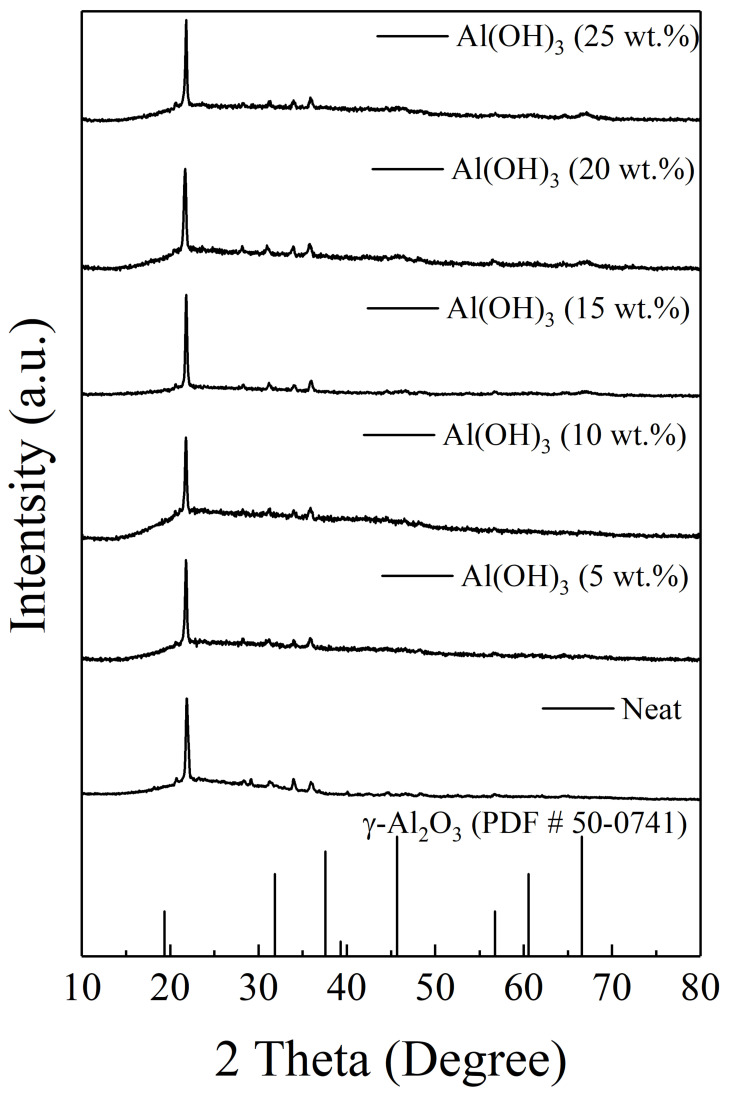
XRD images of intumescent flame-resistant coatings after thermal heating with the optimal ratio of sodium silicate, ammonium polyphosphate, pentaerythritol, and additional 5, 10, 15, 20, and 25 wt.% of Al(OH)_3_, respectively.

Al(OH)_3_ is often utilized and is well-known for its synergistic effect as an endothermic and phase-transformed material in improving flame-retarding abilities. Its anti-combustible mechanism is that it decomposes into water moisture or vapor to become Al_2_O_3_ at higher temperatures, which can delay flame propagation. Figure 8 shows numerous pores that gradually emerged in the carbon char layer narrow after thermal exposure, which simultaneously reinforce the rigidity and improve compactness. The data summarized in Figure 9 indicate that the maximal expansion ratio of intumescent flame-resistance coating at 5 wt.% of Al(OH)_3_ is enhanced by the presence of water stream. The inclusion of the Al(OH)_3_ is beneficial to the formation of Al_2_O_3_, except for the increasing expansion ratio upon heating. Hardness is found to be reversely proportional to expansion ratio, which declines from the highest value of 58.4 HD with no Al(OH)_3_ to that of 34.14 HD with 5 wt.% ratio, and then both HDs raise slowly. The main reason for this is that in the ambient, more than 5 wt.% of Al(OH)_3_ fill the voids within the coating, eventually leading to an increase in hardness. Interestingly, more Al(OH)_3_ can also weaken the pull-off strength, probably due to an influential decline in -Si-O-Fe bonding. The XRD patterns in Figure 10 verify the formation of γ-Al_2_O_3_ (JCPDS-PDF #50-0741) at high temperatures, which is produced after 1200 °C. The presence of the γ-Al_2_O_3_ as a void-filling agent may prevent the voluminous carbon-char layer from cracking, and simultaneously withstand the invasion of flame.

### 3.4. Effects of Expandable Graphite on the Physical Properties and Flame Testing of Intumescent Flame-Resistant Coating Materials

**Figure 11:**
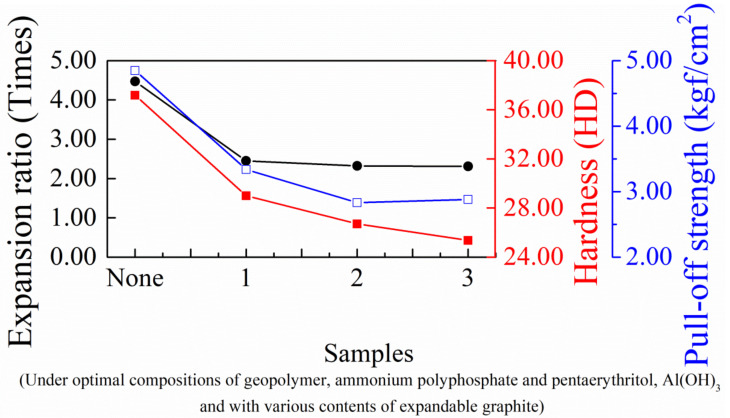
Physical properties of intumescent flame-resistant coatings after thermal heating with optimal ratio of sodium silicate, ammonium polyphosphate, pentaerythritol and various contents of expandable graphite.

Despite the efficient, high flame-resistance and low thermal conduction of the coating, the viable way to improve the intumescent coating as much as possible is to further avoid heat transfer from the outer environment to the inner steel substrate in the flame testing. This can be accomplished by using expandable graphite to fill the inner part of the steel substrate with air, isolating it from the flame. The physical properties of expansion ratio, hardness and pull-off strength in Figure 11 all decrease with expandable-graphite loading over the high-temperature heating in the oven, which is puzzling. In terms of our available data, some additional factors are probably associated with the extent of the expansion ratio, such as the decreasing amount of sodium silicate binder in the composites, which can reduce the chemical interaction between the sodium silicate binder and expandable graphite. The introduction of fragile expandable graphite may effectively destroy the mechanical properties of the matrix.

Nevertheless, the literature reported that heating rate [36] can control the degree of expansion ratio. However, Duquesne et al. [37] suggest that with the addition of up to 25 wt.% of expandable graphite in the matrix, the measured heat transfer coefficient can be minimized to be 0.21 ± 0.02 W/m K at 400 °C, along with an increase in the expansion ratio. Their literature persuaded us to add a small amount of expandable graphite (1 wt.%) as the intumescent improver in the formula.

**Figure 12:**
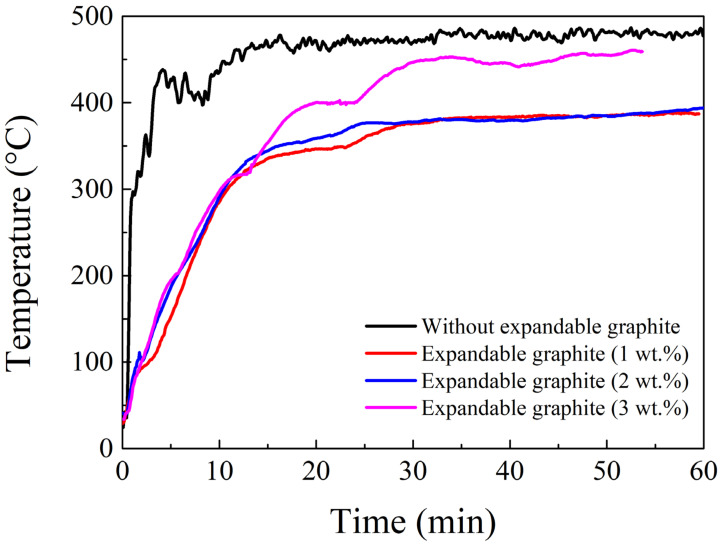
Flame testing of intumescent flame-resistant coatings with the optimal ratio of sodium silicate, ammonium polyphosphate, pentaerythritol, Al(OH)_3_ and 1~3 wt.% loading of expandable graphite, respectively.

**Figure 13:**
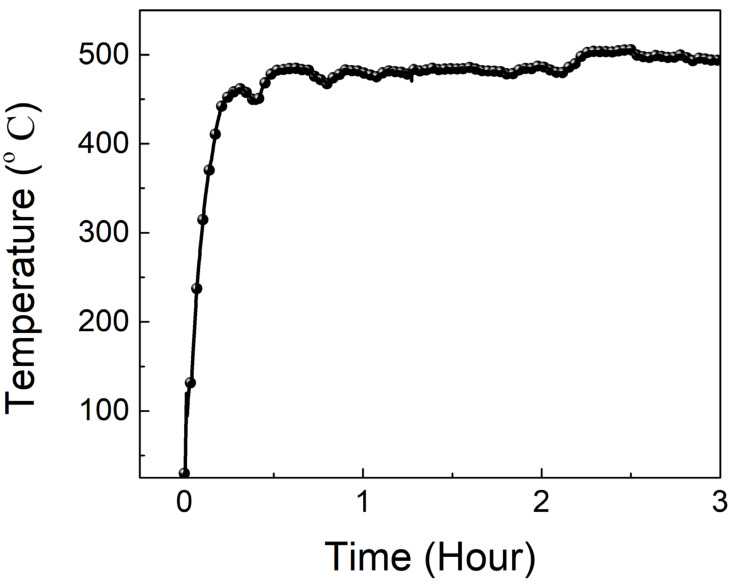
Back surface temperature vs. time of the three-hour flame testing for intumescent flame-resistance coating with the obtained optimal compositions.

To investigate the effect of expandable graphite, flame testing was conducted by a pilot flame with the obtained optimal ratio of the coating and additional 1~3 wt.% loading of expandable graphite. The results in Figure 12 demonstrate that the temperature of flame testing for a sample without expandable graphite steeply rises, compared to samples with expandable graphite. Within an hour of flame testing, the temperature difference hit almost 100 °C between samples with and without the addition of expandable graphite. This temperature drop comes from the contribution of the layer-by-layer structure in the expandable graphite, offering a remarkable barrier effect and delaying heat transfer. This kind of heat-delaying effect can cooperate with ammonium polyphosphate and pentaerythritol to establish a new type of synergist system. In terms of our results, although expandable graphite has many merits, such as a layer-by-layer structure, to improve the flame-retarding ability, overloading (>2 wt.%) expandable graphite in the matrix might destroy the mechanical properties of the flame-retardancy coating based on our results. Therefore, for long flame testing, temperature at the backside of steel substrate is higher, with 3 wt.% expandable graphite compared to 1 or 2 wt.%. The temperature of the backside of steel substrate is measured at 387.1 °C using the optimal ratio of sodium silicate—metakaoline (at a weight ratio of 9:1)—ammonium polyphosphate and pentaerythritol, aluminum hydroxide (3, 3, 10 wt.%)—expandable graphite (1 wt.%). Table 1 lists the expansion ratio data plus the contribution from expandable graphite, which concurs with the flame testing. Furthermore, Figure 13 shows that the inorganic intumescent fire-retarding coating can keep the temperature of the back surface of testing steel at 506.3 °C during a three-hour period of combustibility, suggesting that it can provide a three-hour rated protection for the steel structure in a combustible environment.

## 4. Conclusions

The flame-resistant abilities can be enhanced by the inorganic intumescent coating containing sodium silicate, metakaolin, ammonium polyphosphate, pentaerythritol, aluminum hydroxide and expandable graphite, which play individual roles as a ceramizable matrix, flame-retardant filler, heat-absorber and intumescence-improver, respectively. The physical properties, including the expansion ratio, hardness and pull-off strength of the inorganic intumescent coating, were all investigated. Behaving as a kind of geopolymer with a sodium silicate to metakaolin weight ratio of 9:1, it can be used as a flame-retarding material with 16.0 times the expansion ratio, 38.7 HD of hardness and 5.7 kgf/cm^2^ of pull-off strength. The flame-retardant filler acts as a carbon-char layer, providing thermal stability at high temperatures. An excess of ammonium polyphosphate can react with sodium silicate to produce Na_3_PO_4_. The inclusion of Al(OH)_3_ as a promoter and filler can significantly improve the expansion ratio. With the addition of expandable graphite, hourly flame testing reaches 387.1 °C in the backside of steel substrate, along with 4.7 times the expansion ratio. The backside temperature can also be maintained at 506.3 °C for three-hour flame testing on the steel substrate, ascribing to both a better thermal insulation and improved flame resistance.

## Figures and Tables

**Table 1:** Expansion ratio and thickness of intumescent flame-resistant coating before (L1) and after (L2) direct flame testing.

Sample	L1 (mm)	L2 (mm)	Expansion Rate (Times)
Neat	5.0	18.1	3.6
expandable graphite (1 wt.%)	5.0	23.5	4.7
expandable graphite (2 wt.%)	5.0	25.2	5.1
expandable graphite (3 wt.%)	5.0	21.9	4.3
